# Modulation of Tubular pH by Acetazolamide in a Ca^2+^ Transport Deficient Mice Facilitates Calcium Nephrolithiasis

**DOI:** 10.3390/ijms22063050

**Published:** 2021-03-17

**Authors:** Eugenia Awuah Boadi, Samuel Shin, Samuel Yeroushalmi, Bok-Eum Choi, Peijun Li, Bidhan C. Bandyopadhyay

**Affiliations:** 1 Calcium Signaling Laboratory, Research Service, Veterans Affairs Medical Center, 50 Irving Street, NW, Washington, DC 20422, USA; eawuahbo@terpmail.umd.edu (E.A.B.); samuel.shin@va.gov (S.S.); yeroush@gmail.com (S.Y.); bc586@georgetown.edu (B.-E.C.); pjl72@hotmail.com (P.L.); 2 Division of Renal Diseases & Hypertension, Department of Medicine, The George Washington University, Washington, DC 20037, USA; 3 Department of Biomedical Engineering, The Catholic University of America, 620 Michigan Avenue NE, Washington, DC 20064, USA

**Keywords:** acetazolamide, renal tubular pH, proximal tubule, Ca^2+^ signaling, oxidative stress, inflammation, fibrosis, apoptosis, calcium nephrolithiasis, urinary stones

## Abstract

Proximal tubular (PT) acidosis, which alkalinizes the urinary filtrate, together with Ca^2+^ supersaturation in PT can induce luminal calcium phosphate (CaP) crystal formation. While such CaP crystals are known to act as a nidus for CaP/calcium oxalate (CaOx) mixed stone formation, the regulation of PT luminal Ca^2+^ concentration ([Ca^2+^]) under elevated pH and/or high [Ca^2+^] conditions are unknown. Since we found that transient receptor potential canonical 3 (TRPC3) knockout (KO; -/-) mice could produce mild hypercalciuria with CaP urine crystals, we alkalinized the tubular pH in TRPC3-/- mice by oral acetazolamide (0.08%) to develop mixed urinary crystals akin to clinical signs of calcium nephrolithiasis (CaNL). Our ratiometric (λ340/380) intracellular [Ca^2+^] measurements reveal that such alkalization not only upsurges Ca^2+^ influx into PT cells, but the mode of Ca^2+^ entry switches from receptor-operated to store-operated pathway. Electrophysiological experiments show enhanced bicarbonate related current activity in treated PT cells which may determine the stone-forming phenotypes (CaP or CaP/CaOx). Moreover, such alkalization promotes reactive oxygen species generation, and upregulation of calcification, inflammation, fibrosis, and apoptosis in PT cells, which were exacerbated in absence of TRPC3. Altogether, the pH-induced alteration of the Ca^2+^ signaling signature in PT cells from TRPC3 ablated mice exacerbated the pathophysiology of mixed urinary stone formation, which may aid in uncovering the downstream mechanism of CaNL.

## 1. Introduction

Renal tubular acidosis (RTA) is characterized by defective renal acid-base regulation due to an inability to excrete hydrogen ions (H^+^) [1]. While distal tubular RTA is associated with reduced urinary acid secretion, proximal tubular (PT) RTA results due to impaired bicarbonate (HCO_3_^−^) reabsorption, ensuing in the decline of serum HCO_3_^−^ and alkaline pH at the tubular lumen [2]. Such condition in PT can evoke calcium phosphate (CaP) crystallization, because, in addition to increasing extracellular Ca^2+^ concentration ([Ca^2+^]_o_), the ambient pH has a profound effect on the supersaturation of Ca^2+^, and the crystallization of CaP is strongly favored by a pH of 6.5 or greater [3]. The solubility of CaP crystals is also pH-dependent and is greatly increased at acidic pH (<pH 6.2). In hypercalciuric patients with calcium stones, an incomplete renal PT acidosis has been characterized by abnormal bicarbonaturia [4]. Especially if other risk factors such as hypercalciuria are present, the transient bicarbonaturia in these patients promotes favorable conditions to form CaP crystals, possibly at the tip of the loop of Henle (LOH), where luminal pH normally increases to 7.3 [5]. The presentation of relatively high urine pH and low urine citrate with a normal serum HCO_3_^−^ concentration in the patient are quite typical of CaP stone formers [6]. Moreover, a higher urine pH distinguishes CaP stone formers from calcium oxalate (CaOx) ones, whereby CaP stone-formation could be recurrent [7]. Thus, the interplay between PT Ca^2+^ regulation and the rise in PT luminal pH could be fundamental to the downstream mechanism and the regulation of CaP or CaP+CaOx mixed stone formation.

Notably, patients with RTA, as well as those who are being treated with acetazolamide (Acz), a diuretic, commonly prescribed for epilepsy, heart failure, and glaucoma, show an increased risk of CaP stone formation [8,9]. Acz’s mechanism of action to block carbonic anhydrase (CA) II can subsequently produce similar physiological symptoms to proximal RTA. CAII, which is primarily localized in the PT cells, catalyzes the interconversion of CO_2_ and H_2_O to HCO_3_^−^ [10]. Inhibition of CAII suppresses H^+^ excretion from PT cells, leading to alkaline urine and subsequent systemic acidosis [11]. Furthermore, such reduced H^+^ excretion further decreases the action of an apical Na^+^/H^+^ exchanger, resulting in higher Na^+^ excretion, which draws water into the luminal space via osmosis and creates the diuretic effect exhibited by Acz [12]. Consequently, Acz can promote kidney stone formation by increasing the supersaturation of Ca^2+^ in a similar manner to RTA [9]. In patients, who present with consistently alkaline urine, as when afflicted with RTA or upon treatment with Acz, minimal CaP dissolution occurs, and its supersaturation increases rapidly at pH 6 to 7 [13]. Additionally, metabolic acidosis induced by Acz can activate osteoclasts as well as inhibit renal tubular Ca^2+^ reabsorption leading to excess Ca^2+^ excretion [14]. Importantly, Acz can also cause hypocitrauria, even further exacerbating the formation of calcium stones, since citrate is a Ca^2+^ chelator which reduces free Ca^2+^ in the tubular filtrate [15,16]. However, the molecular mechanism of tubular alkalization-induced stone-forming pathophysiology is not clear.

Predisposing factors caused by metabolic abnormality could also significantly increase the risk of calcium stone formation, such as idiopathic hypercalciuria which is present in most calcium stone formers [8,17]. Recently, we found that transient receptor potential channel 3 (TRPC3) channel knockout (KO; -/-) mice exhibited elevated urinary Ca^2+^, and CaP calcinosis at the LOH region [18]. TRPC3 is also localized further downstream in distal tubular cells, but our evidence, which depicts the presence of CaP crystals at the LOH in TRPC3 KO mice, reasonably indicates the strong influence of TRPC3 mediated PT Ca^2+^ transport in such process [18]. We proposed that the deficiency in PT Ca^2+^ transport in TRPC3 KO mice can be linked to tubular Ca^2+^ supersaturation when compared to the wild type (WT) counterpart. However, our previous findings have not shown the human-like condition found among the calcium stone formers, which may require the rise in pH within the PT luminal fluid [18]. Such initial CaP (apatite) crystallization could be the root cause of CaP+CaOx mixed stone formation downstream in the distal part of the nephron [8], which may have promoted heterogeneous nucleation further downstream in the papillary region [19]. Therefore, to understand the mechanism of mixed stone-formation, we escalated the filtrate pH by oral Acz treatment in our moderately hypercalciuric (TRPC3-/-) mice to create a human-like stone-forming pathophysiology in vivo to understand the underlying mechanism of CaNL.

## 2. Results

### 2.1. Urine Alkalization in Mice Increases Ca^2+^ Entry in PT Cells

**Figure 1:**
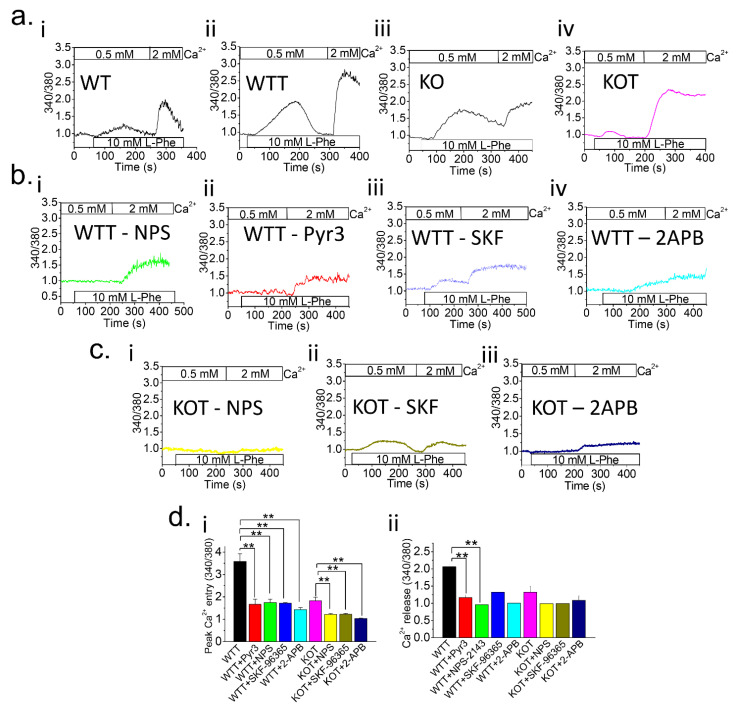
Higher luminal pH in TRPC KO PT cells enhances Ca^2+^ entry. [Ca^2+^]_i_ measurements were performed on Fura-2 loaded (**a**) control PT cells and (**b,c**) PT cells with NPS-2143 (NPS; 1 μM), Pyr3 (3 μM), SKF-96365 (SKF; 10 μM) or 2-APB (12 μM) inhibitors from WT, Acz-treated WT (WTT), TRPC3-/- (KO), or Acz-treated KO (KOT) mice. Prior to experiments, PT cells were placed in 0.5 mM Ca^2+^ bath and L-Phenylalanine (L-Phe; 10 mM) was applied. Subsequently, 2 mM Ca^2+^ was applied to replenish [Ca^2+^]_o_. Fura-2 trace measurements were plotted for (**ai**) WT, (**aii**) WTT, (**aiii**) KO, and (**aiv**) KOT; WTT PT cells with (**bi**) NPS, (**bii**) Pyr3, (**biii**) SKF, and (**biv**) 2-APB inhibitors; and KOT PT cells with (**ci**) NPS, (**cii**) SKF, and (**ciii**) 2-APB inhibitors. (**di**,**dii**) Peak Ca^2+^ entry and Ca^2+^ release were quantified with representative bar diagrams in means ± SEM, respectively. Each experiment was performed four times. **, *p* < 0.01.

To understand the Ca^2+^ signaling profile in PT cells during Acz-induced alkalization, we measured Ca^2+^ transients while activating calcium sensing receptor (CaSR) in PT cells from WT, Acz treated WT (WTT), TRPC3 KO and Acz treated TRPC3 KO (KOT) mice by allosteric activation of CaSR by L-Phenylalanine (L-Phe) application (Figure 1a). In WT PT cells, stimulation of CaSR by L-Phe in presence of nominal [Ca^2+^]_o_ elicited a small release followed by a rapid rise in [Ca^2+^]_i_ due to Ca^2+^ entry upon addition of excess [Ca^2+^]_o_, which was then steadily declined (Figure 1a,d). Such activation in WTT PT cells produced a relatively large Ca^2+^ release and Ca^2+^ entry compare to WT, however the decline in Ca^2+^ entry was sluggish (Figure 1a,d), perhaps due to greater activation of CaSR by higher luminal pH. As expected, in KO cells, Ca^2+^ entry was significantly reduced due to the absence of TRPC3 (Figure 1a), whereas, much larger responses were found in KOT compared to the KO, and were much sustained compared to the WTT group (Figure 1a), possibly by a non-TRPC pathway. Interestingly, Ca^2+^ release after Acz treatment revealed diametrically opposing effects depending on the presence or absence of TRPC3 (Figure 1a,d), which can be explained by the fact that, in the absence of TRPC3, [Ca^2+^]_i_ may be accumulated in KOT PT cells, compared to the WTT counterpart.

To delineate the Ca^2+^ entry pathway in both WTT and KOT PT cells, we tested several inhibitors: NPS-2143 (for CaSR), Pyr3 (for TRPC3), SKF-96365 (for TRP channel), and 2-Aminoethoxydiphenyl borate (2-APB; for inositol triphosphate (IP_3_) receptor) [20, 21, 22, 23]. In the presence of NPS-2143, Ca^2+^ release in both WTT and KOT PT cells was completely inhibited (Figure 1b,d), while the Ca^2+^ entry of KOT cells was completely inhibited, unlike that of the WTT cells. In turn, application of Pyr3 on WTT cells had a similar inhibitory effect on Ca^2+^ release and Ca^2+^ entry (Figure 1b,d), confirming the involvement of TRPC3 in both Ca^2+^ entry and release. Inhibition by SKF-96365 was prominent in both WTT and KOT after L-Phe induction for Ca^2+^ release and Ca^2+^ entry (Figure 1b,d). It is worth noting that SKF-96365 is also known to inhibit the SOCE pathway, which can attribute to possible indirect effects [24]. Interestingly, IP_3_ receptor inhibition by 2-APB has considerably decreased both Ca^2+^ release and entry in WTT and KOT cells (Figure 1b,d).

### 2.2. Mode of Ca^2+^ Entry Switches the from Receptor-Operated Ca^2+^ Entry (ROCE) to Store-Operated Ca^2+^ Entry (SOCE) in PT Cells Following Urine Alkalization

**Figure 2:**
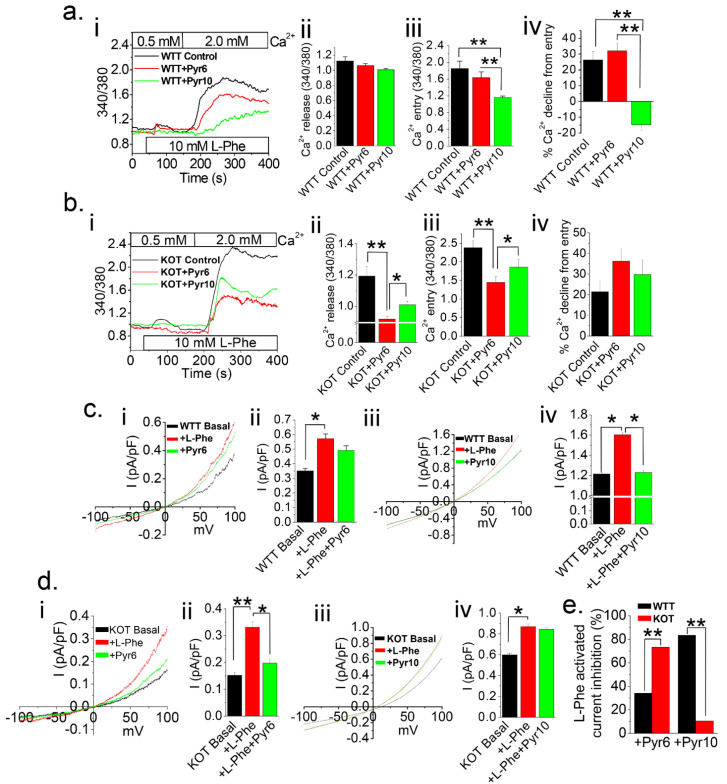
Ca^2+^ entry pathway in PT cells is contingent upon presence of TRPC3 in higher PT luminal pH conditions. Ratiometric [Ca^2+^]_i_ measurements were performed and plotted on Fura-2 loaded PT cells from Acz treated mice (**a**) WT (WTT) and (**b**) KO (KOT) PT cells with no inhibitors (WTT Control or KOT Control), Pyr6 (3 μM), or Pyr10 (3 μM). Prior to experiments, PT cells were placed in a 0.5 mM Ca^2+^ bath and L-Phenylalanine (L-Phe; 10 mM) was applied. Subsequently, 2 mM Ca^2+^ was applied to replenish [Ca^2+^]_o_. (**aii**,**bii**) Peak Ca^2+^ release**,** (**aiii**,**biii**) Peak Ca^2+^ entry, and (**aiv**) Percent Ca^2+^ decline from entry were quantified into bar diagrams in means ± SEM. (**c**,**d**) Single cell patch clamp voltage sweeps from −100 to +100 mV were performed on WTT or KOT PT cell. L-Phe (10 mM) was applied to induce current and (**ci**,**cii**,**di**,**dii**) Pyr6 (3 μM) or (**ciii**,**civ**,**diii**,**div**) Pyr10 (3 μM) was applied as a current blocker. (**cii**,**civ**,**dii**,**div**,**e**) Average current density at +100 mV and L-Phe activated current inhibition percentage were quantitated into means ± SEM, respectively. Experiments were performed four times. *, *p* < 0.05; **, *p* < 0.01.

CaSR activation can trigger both ROCE and SOCE pathways in PT cells [18], thus, we also exposed the WT and WTT PT cells with Pyr6, an SOCE blocker, or Pyr10, an ROCE blocker to delineate the exact contribution of those Ca^2+^ signaling pathways in triggering [Ca^2+^]_i_ entry in PT cells during induced alkalization [25]. Notably, Ca^2+^ release upon activating the CaSR has corresponded with the Ca^2+^ release pathway in PT cells [18], which is indicative of SOCE pathway activation (Figure 2a). We found that Ca^2+^ release among WTT cells did not change in presence of Pyr6 or Pyr10 compared to the control (Figure 2a). When comparing Ca^2+^ entry inhibitions by Pyr6 or Pyr10 in WTT cells, Pyr10 has inhibited most of the Ca^2+^ entry compared to Pyr6 (Figure 2a). Evidently, Pyr6 and Pyr10 both significantly reduced the Ca^2+^ entry compared to the control (Figure 2a). Moreover, activating CaSR in KOT cells exhibited a slightly decreased Ca^2+^ entry compared to that of the WTT (Figure 2a,b). Interestingly, when we compared the Ca^2+^ entries of KOT cells in presence of Pyr6 or Pyr10, the former has blocked approximately two-thirds of the entry, which was greater than one-third of the entry blocked by the latter, which demonstrated that the primary Ca^2+^ entry pathway in absence of TRPC3 upon Acz treatment is mediated by SOCE (Figure 2b). Since the TRPC3 channel is not expressed in these KOT cells, some other pathway involving IP_3_ may be involved.

To confirm our findings, we performed electrophysiological experiments by whole-cell patch clamp recordings on PT cells from WTT and KOT mice (Figure 2c–e). Application of L-Phe has elicited a TRPC-like current activation in both WTT and KOT cells (Figure 2c,d). Such a current in WTT PT cells was almost completely blocked by Pyr10, whereas the SOCE blocker, Pyr6, did not reduce it, indicating that similar to WT PT cells, ROCE remains the major pathway in WTT PT cells (Figure 2c,e). In contrast, in KOT PT cells, Pyr10 application did not block any CaSR-induced current activation, and Pyr6 inhibited most of the activated current (Figure 2d,e), suggesting that in absence of TRPC3 when PT cells exposure to alkaline condition, the mode of Ca^2+^ entry has been reversed through the activation of SOCE pathway.

### 2.3. Urine Alkalization Enhances HCO_3_^−^ Related Current Activity in PT Cells 

**Figure 3:**
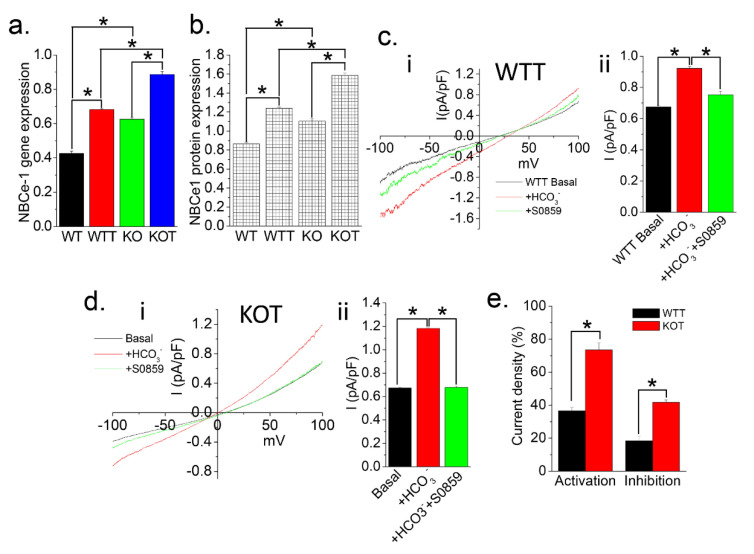
NBCe1 expression and function are enhanced in TRPC3 KO PT cells in higher luminal pH conditions. (**a**) mRNA and (**b**) protein expressions of NBCe1 in PT cells from WT, Acz-treated WT (WTT), TRPC3 KO (KO) and Acz treated TRPC3 KO (KOT) mice were quantitated into bar diagrams. (**c**,**d**) Whole cell patch clamp voltage sweeps from −100 to +100 mV were quantitated into I-V curves and average current density at +100 mV was calculated. Bicarbonate (0.3 mM) was applied to induce current density and S0859 (50 μM) was applied as a current blocker. (**a**,**b**,**ci**,**cii**,**di**,**dii**) NBCe1 gene and protein expression, and average current density at +100 mV were quantitated into means ± SEM, respectively. (**e**) Percentage current density activation (bicarbonate) and inhibition (S0859), respectively, were quantitated into means ± SEM. Experiments were performed four times. *, *p* < 0.05. Gene and protein expressions are normalized to GAPDH and β-actin respectively.

Sodium-bicarbonate exchanger (NBCe1) in PT cells plays a prominent role in the regulation of intracellular HCO_3_^−^, which is critical to the regulation of PT luminal pH [26]. Thus, we looked at the expression of NBCe1 in those PT cells from treated (WTT and KOT) and untreated (WT and KO) mice and found an increase in mRNA (Figure 3a) and protein (Figure 3b) expression patterns for both WTT and KOT groups, suggesting the functional compensation due to renal acidosis caused by the Acz treatment (Figure 3a,b). Next, we performed electrophysiological experiments on WTT and KOT PT cells, using HCO_3_^−^ as the agonist and S0859, an inhibitor of NBCe1, to assess the alkalization-induced modulation of NBCe-1 currents in PT cells [27]. After establishing the basal current, we applied 0.3 mM HCO_3_^−^ to the PT cells, which induced the current and was subsequently blocked by S0859 (Figure 3c).

Notably, the I-V curve displayed a slight inwardly rectified current (Figure 3c), similarly to the Ca^2+^ release activated channel (CRAC) current, which may be implicated by the Acz treatment in presence of TRPC3. However, in the case of the KOT cells, the current resembles less of a CRAC current and more of an outwardly rectifying current (Figure 3d). Interestingly, while comparing both PT cell groups (WTT vs. KOT), the current activation by HCO_3_^−^, was significant in both cases, and appeared more prominent for the KOT cells than the WTT cells (Figure 3e). Since most of the HCO_3_^−^ handling involves NBCe1 in PT cells [26], S0859 inhibition confirms this activity (Figure 3c,d). As a result, although in WTT cells most of the current has been inhibited, almost all the current activated by HCO_3_^−^ has been blocked by S0859 in the case of KOT cells (Figure 3c,d). Overall, these results suggest that KOT cells exhibit higher NBCe-1 activity than that of the WTT cells.

### 2.4. Acz-Induced Alkaluria Determines the Type (CaP or CaP+CaOx) of Calcium Stone 

**Figure 4:**
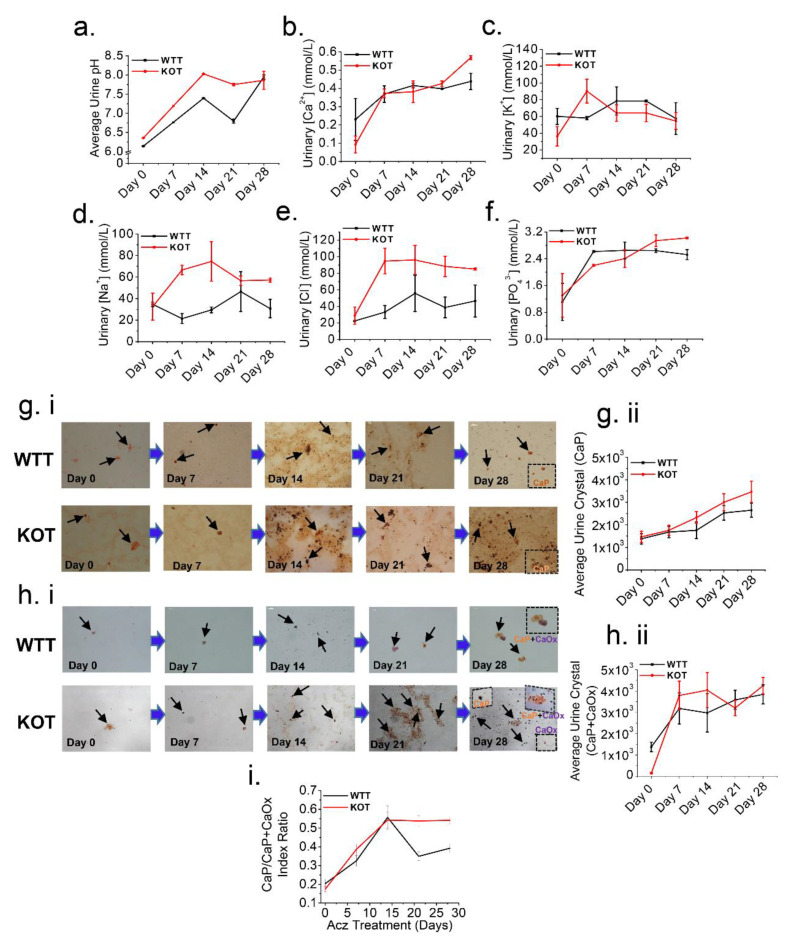
Higher luminal pH induced by Acz treatment induces CaP crystals formation. Representative line graphs of (**a**) Urine pH, urine electrolyte excretion levels (**b**) Ca^2+^, (**c**) K^+^, (**d**) Na^+^, (**e**) Cl^−^ and (**f**) PO_4_^3−^, collected on day 0, 7, 14, 21 and 28 of the treatment periods immediately after 24 h urine collection. (**a**,**b**) and (**d**–**f**) Urinary pH, Ca^2+^, Na^+^, Cl^−^ and PO_4_^3−^ clearance progressively increased throughout the treatment period, particularly for the KOT group. (**c**) Urine K^+^ on the other hand showed a reduction in clearance for both the untreated and treated groups throughout the treatment period. All urine measurements were normalized by creatinine. (**gi**) Alizarin Red (AR) pH 4.3 (stains CaP crystals) and (**hi**) pH 6.8 (stains both CaOx and CaP+CaOx crystals) to differentially visualize calcium crystals present in the urine. Black arrows show urine crystals (CaP and CaP+CaOx). (**gii**,**hii**) representative line graphs for both WTT and KOT groups showing progressive increase in CaP and CaP+CaOx urine crystals respectively following Acz treatment. (**i**) CaP to CaP/CaOx index ratios throughout Acz treatment period which compared WTT and KOT mice groups were quantitated into means ± SEM. Experiments performed *n = 4* times.

Since TRPC3 KO mice are moderately hypercalciuric [18], we treated them with Acz to induce in vivo CaP stone formation. To examine how the treatment can influence the regulation of clinically relevant urine electrolytes, we performed ionic measurements of 24 h mice urine. Our data show that urinary levels of Ca^2+^, Na^+^, K^+^, PO_4_^3−^, and Cl^−^ showed a progressive increase in excretion in the KOT group compared to the WTT by Day 28 (Figure 4a–f), except for K^+^ which showed a slight reduction in excretion levels in those treatment groups (Figure 4c). Serum pH of WTT and KOT mice revealed lower serum pH levels compared to the WT and KO groups (Figure S1a). Serum electrolyte levels for Ca^2+^, Na^+^ and Cl^−^ (Figure S1b,d,e) were relatively similar among all groups while serum K^+^ and PO_4_^3−^ levels consequently increased in both the WTT and KOT groups (Figure S1c,f). Urine and serum creatinine levels did not change throughout most of the treatment, ruling out changes in clearance rate (Figure S1g,h). Overall, these results lend support for the increase in CaP and the resulting mixed crystal formation (Figure 4g,h), which is particularly visible in KOT mice. Most notably, by the end of the treatment, more CaP crystal formation was observed in KOT mice compared to the WTT group, because more CaP may have been formed in absence of TRPC3 (Figure 4i). While the urine volumes due to diuretic effect of Acz during the treatment period in both WTT and KOT were visible (Figure S1i) and its alkaline effect dictated the stone phenotype (CaP/CaP+CaOx). Serum PTH (Figure S1j) and dietary intake did not change among all groups (Figure S1k). Notably, the expressions of transepithelial markers SLC26a, NaPiiIIa and PMCA1 were similar in all groups tested, except NCX1 showing increased expression in the KO and KOT groups (Figure S2).

### 2.5. Alkalization-Induced Calcification Was Upregulated in Absence of TRPC3

**Figure 5:**
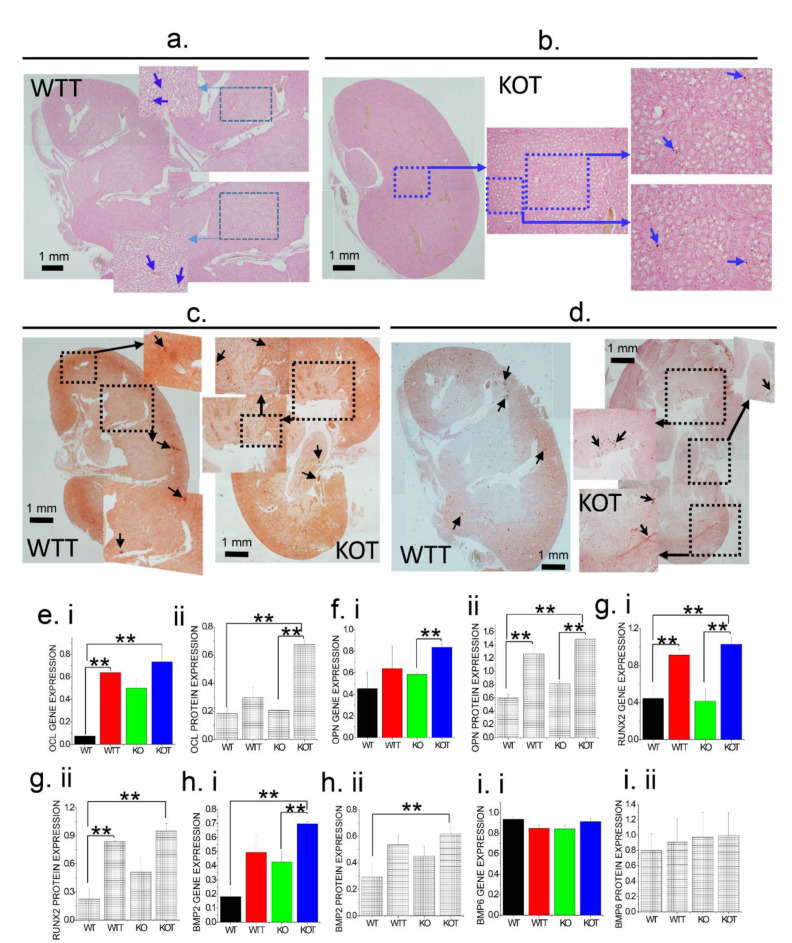
Histological and molecular characterization of kidney calcification and stone formation following Acz treatment. Evidence of kidney calcification and stone formation by examining kidney tissues and its underlying molecular analysis through gene/protein expression of calcification markers. (**a**,**b**) Representative Von Kossa staining (for CaP) of whole kidney sections of WTT and KOT showing calcified regions of the kidney indicated by blue arrows. (**c**) Alizarin Red (AR) staining of whole WTT and KOT kidney sagittal sections of AR pH 4.3 staining shows CaP stones present in the LOH region of whole kidney while (**d**) AR pH 6.8 shows CaP+CaOx (mixed) stones in the calyx region of WTT and KOT whole kidney. Gene/Protein expression analysis providing evidence PT cell calcification in treated mice are shown by expression profiles of known calcification markers; (**ei**,**eii**) OCL, (**fi**,**fii**) OPN, (**gi**,**gii**) RUNX2 and (**hi**,**hii**) BMP2, which are significantly higher for the treated groups especially for KOT compared to the controls (WT and KO), except for (**ii,iii**) BMP6 which did not show any significant difference between the treated and untreated groups. (**e**–**i**) Bar graphs quantifications are means ± SEM, (*n* = 3); **, *p* < 0.01. All gene and protein expressions are normalized to GAPDH and β-actin respectively.

Analyses of crystal formation revealed that following alkaluria induced by Acz treatment, significant crystal formation occurred in both WTT and KOT mice. We therefore determined the degree of calcification in vivo in kidneys of our treated groups (WTT and KOT). Von Kossa staining results of kidney sagittal sections revealed that KOT groups showed more calcification than in WTT groups (Figure 5a,b) while CaP (Figure 5c) and CaP+CaOx (Figure 5d) crystals in the kidney, predominantly located in the LOH and calyx regions, were increased particularly in the KOT group following Alizarin Red (AR) pH 4.3 or 6.8 staining respectively. We further corroborated our findings through gene expression analysis of calcification genes and protein analysis in PT cells of our treated mice (WTT and KOT) by comparatively analyzing our results with control groups (WT and KO). Our results show that analysis of gene and protein expression of Osteocalcin 2 (OCL2), Osteopontin 4 (OPN-4), and Runt-related transcription factor 2 (RUNX2) were significantly higher in both the WTT and KOT groups when compared to WT and KO groups (Figure 5e–g). Similarly, in the expression of profiles of bone morphogenic protein (BMP), BMP-2 showed a similar expression (Figure 5h) while BMP-6 showed no difference between the treated and control groups (Figure 5i). From our results, we show that PT calcification is more pronounced under conditions of higher luminal pH, particularly when coupled with the absence of TRPC3.

### 2.6. Alkalization Induced by Acz Results in Inflammatory and Fibrotic Responses in PT Cells 

**Figure 6:**
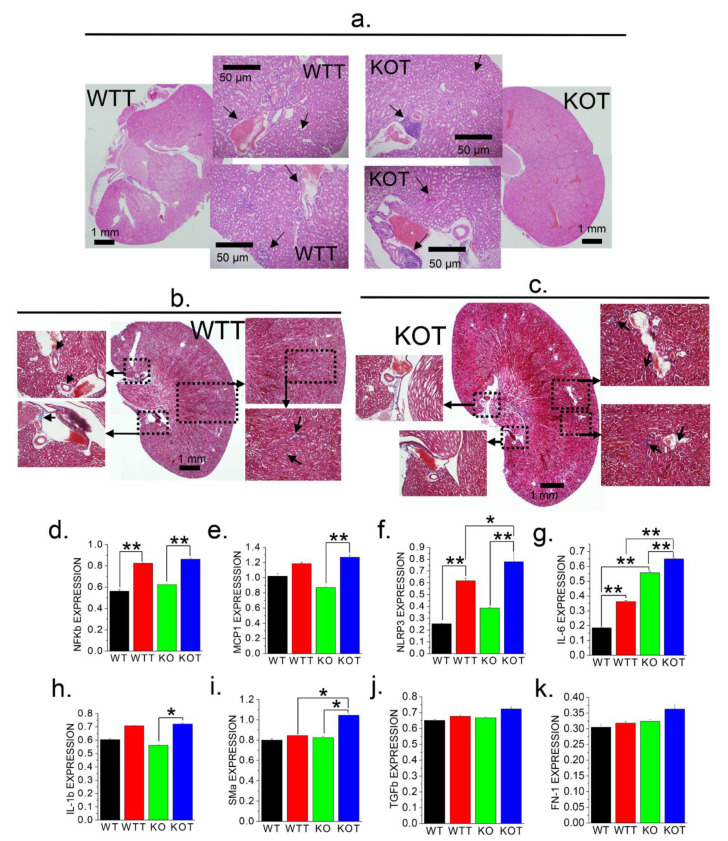
Histological and molecular characterization of kidneys for fibrosis and inflammation following Acz treatment**.** Supportive evidence of Acz-induced fibrotic and inflammatory induction with of expressions of genetic markers of fibrosis and inflammation in PT cells. (**a**) Representative H&E staining of KOT and WTT mice kidney sections showing regions of inflammation in the kidney. (**b**,**c**) WTT and KOT mice kidney sections of Masson’s Trichrome staining shows fibrotic regions of the kidney. Corroborative evidence through gene expression analysis for fibrotic and inflammatory markers (**d**) NFκβ, (**e**) MCP1, and (**f**) NLRP3 in WT, WTT, KO, and KOT mice PT cells which are significantly higher in the treated groups especially for KOT, compared to the controls. Genetic expression profiles of (**g**) IL-6, (**h**) 1L-1β, (**i**) SMα, (**j**) TGFβ and (**k**) FN-1 in WT, WTT, KO, KOT mice PT cells; except in TGFβ and FN-1, KOT groups yielded higher expressions. (**d**–**k**) Bar graphs are means ± SEM from three separate experiments. *, *p* < 0.05; **, *p* < 0.01. All gene expressions are normalized to GAPDH.

To understand the crosstalk between RTA and inflammation, we examined inflammatory signatures during elevated tubular pH in our TRPC3 KO mice model [28,29]. While inflammatory signals have been shown in PT cells following renal injury, we also used our alkalization model to examine the interaction between inflammation and PT cell fibrosis in the progression of renal damage by examining fibrotic markers in PT cells. H&E stained WTT and KOT kidneys displayed areas of inflammation (Figure 6a). However, Masson’s staining indicated some areas of fibrosis (Figure 6b) in kidneys of WTT while more inflammation and fibrosis were observed particularly in the KOT groups (Figure 6a,c). Furthermore, our gene expression analysis revealed genetic expression levels for inflammation markers such as NFkβ, MCP1, NLRP3 and Interleukins (IL): IL-1β, IL-6, in untreated (WT/WTT) and treated Acz-treated (KO/KOT) PT cells (Figure 6d–h). Our results showed that the treated groups revealed a significant upregulation of inflammatory markers especially in the KOT PT cells. Furthermore, studies have shown that NLRP3 activation has been implicated in an inflammasome complex which involves triggering the NF-κB pathway with release of IL-1β all of which may play a significant role in CaNL and eventually in the development of chronic kidney disease [28]. Since we observed the overall upregulation of inflammatory condition following Acz treatment, we were interested to examine the SMa, TGFβ1, and FN-1, as fibrotic marker gene expression (Figure 6i–k) [30,31,32], to understand the pathophysiology and progression of PT cell injury. While TGFβ1 and FN-1 expressions showed only slight increase in the KOT groups (Figure 6j,k), SMa expression was enhanced in KOT group compared to all others (Figure 6i). Overall, such prominent effect in KOT group suggest that TRPC3 may have a protective role in PT cell fibrosis as the tubular lumen increases its alkalinity.

### 2.7. Oxidative Stress Genes in PT Cells Are Upregulated Due to ALKALIZATION

**Figure 7:**
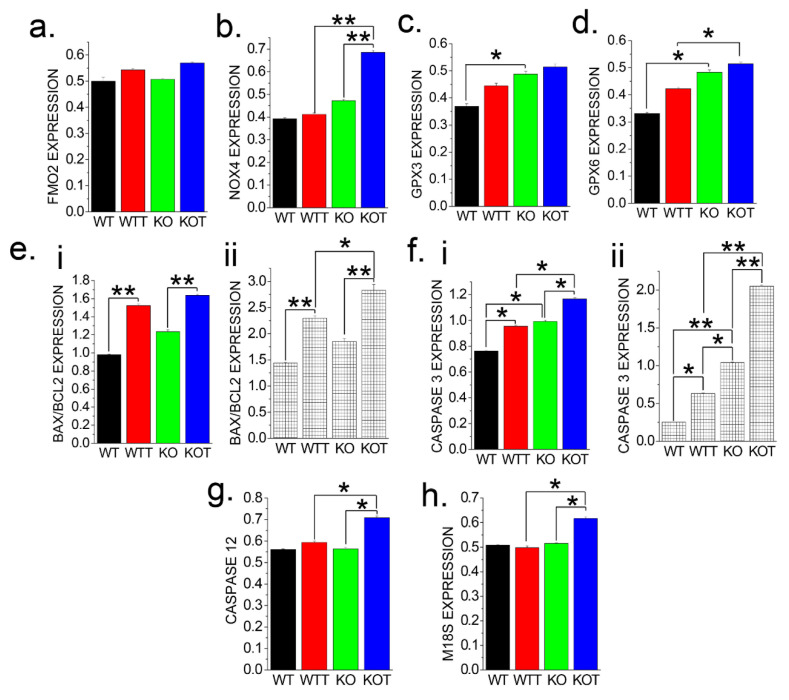
Higher luminal pH induced by Acz increases oxidative stress and promote ER stress-induced apoptosis. Genetic expressions of oxidative stress markers (**a**) FMO2 and (**b**) NOX4, were assessed among WT, WTT, KO and KOT mice PT cells, which show oxidative stress gene, NOX4, expression was significantly increased in the KOT groups compared to all other groups analyzed. (**c**) GPX3 and (**d**) GPX6, markers for protection from oxidative stress, were significantly higher the KOT group compared to all other groups analyzed. Genetic and protein expression for apoptotic markers (**ei**,**eii**) BAX/BCL2 and (**fi**,**fii**) Caspase 3 and (**g**) Caspase 12 were significantly higher for all treated groups especially for KOT group compared to the controls (WT and KO). Evidence of endoplasmic reticulum (ER) stress following Acz treatment through genetic expression levels ER stress marker (**h**) M18S, assessed for WT, WTT, KO, and KOT mice PT cells where the ER stress gene was significantly expressed in the KOT group compared to all other groups analyzed. Experiments were performed three times. (**a**–**h**) Bar graphs are means ± SEM. *, *p* < 0.05; **, *p* < 0.01. All gene and protein expressions are normalized to GAPDH and β-Actin respectively.

PT cells are sensitive to reactive oxygen species (ROS) [33], which can be accumulated during PT acidosis during Acz treatment [34,35]. Therefore, we examined the expressions of oxidative stress marker genes, FMO2 and NOX4 (Figure 7a,b) as well as oxidative protection marker genes, Glutathione peroxidases (GPX) 3 and 6 (Figure 7c,d) in PT cells from untreated (WT and KO) and Acz treated (WTT and KOT) mice. Among the oxidative stress marker genes tested, NOX4 expression was shown to be significantly increased in KOT groups compared to all other groups (Figure 7b). Oxidative protection marker genes (GPX3 and GPX6) exhibited an increased expression amongst all the treated groups (WTT and KOT). However, it is worth noting that the rise in oxidative protective markers could be an action by PT cells to offset the rise in ROS production by adjusting ROS to homeostatic levels. Recent studies have shown that ROS and the resulting oxidative stress play a pivotal role in apoptosis [36,37]. Cell death through apoptosis is usually due to response to changes in the cellular microenvironment such as extra and intracellular pH, which can disrupt cellular functions leading to cell death [38]. Therefore, following Acz treatment, we examined the expressions of apoptotic genes, BAX1, BCL2, Caspase 3 and Caspase 12 (Figure 7e–g) in PT cells. We found that the treated groups, particularly the KOT group, have shown a significant increase in caspase gene expression (Figure 7f,g) while BAX/BCL2 expressions was higher in both WTT and KOT (Figure 7e). We confirmed our results by further protein analysis of the same apoptotic markers (Figure 7e,f) which were also consistent (Figure 7(eii,fii)). In addition, previous studies have shown endoplasmic reticulum (ER)-stress induced apoptosis is dependent on Caspase 3 activation and independent of Caspase 12 [39, 40, 41]. Therefore, expression levels of ER-stress gene, M18S was assessed (Figure 7h). We found that M18S levels was significantly increased in the KOT group while all other groups did not show any significant difference, providing support for possible ER-stress induced apoptosis after Acz treatment in absence of TRPC3. Together, these results support the idea that intracellular acidosis in PT cells, coupled with TRPC3 ablation, can aggravate oxidative stress leading to renal injury and inflammation. All of these are detrimental conditions leading to PT cell death that can form cellular debris and work towards the growth and aggregation of calcium crystals [42].

## 3. Discussion

Acz, a sulfonamide derivative, can lower blood pH and increase HCO_3_^−^ in the urine [43]. There is evidence that Acz can induce urinary calculi through its mechanism of inhibiting CA in the PT and therefore cause hyperchloremic metabolic acidosis leading to higher urinary pH [16,44]. Our use of Acz was to simulate high pH conditions at the kidney tubular lumen by dietary supplementation of Acz and to understand the mechanism of CaP stone formation due to alkaluria. Importantly, in addition to urine alkalization, we used TRPC3 KO mice in this study because these mice are moderately hypercalciuric [18], a condition which is conducive for CaP stone formation. Acz was recently used in experimental models for ischemic reperfusion injury recovery, however its CA inhibition further impairs renal oxygenation [45]. Moreover, Acz has also been proposed as a treatment adjunct for patients with cystine and uric acid stone formation to standard alkalization therapy [46]. However, Acz was reported to be poorly tolerated because of its effect in inducing CaP crystal formation [46]. We looked at the activation of CaSR in this study, since precipitation of CaP are dependent upon the elevation of urinary pH, which is linked to the increased activation of CaSR [47]. Acz treatment did not change the expression of transepithelial markers SLC26a, NaPii-IIa and PMCA1 in PT cells (Figure S2), and only a moderate increase in NCX1 (Figure S2d) expression was observed in both the treated and untreated groups. Such upregulation of NCX1 expression perhaps acted as a preventive mechanism against the PT luminal Ca^2+^ supersaturation [47]. Moreover, alkaline urinary pH can be indicative of a higher luminal PT tubule, as demonstrated in stopped flow microperfusion pH measurements of rats treated with Acz [48]. While the expression of SLC26a did not change, CaP+CaOx crystals were abundantly present in KOT mice urine, and Acz could have influenced the activity of the oxalate transport, which could be done in a future study. In assessing dynamic Ca^2+^ transients, a closer look between the different magnitudes of WT and WTT Ca^2+^ entries reveal that a higher luminal pH in the PT can sensitize the CaSR for greater reabsorption of extracellular Ca^2+^ ions. Interestingly, in our study, Ca^2+^ release between both treated groups (WTT and KOT) is dependent upon the presence of TRPC3. Since CAIV, and to a lesser extent CAII, are inhibited by Acz [49], our treated mice may potentially undergo proximal RTA, entailing failure of HCO_3_^−^ handling, and elevated acidity in the PT cells [50], which can have a role in CaP crystal formation in the downstream segments.

Our previous study found that in WT PT cells, the majority of Ca^2+^ entry pathway is mediated by ROCE through the CaSR-induced TRPC3 activation [18]. Tracking down the pathway using various Ca^2+^ signaling inhibitors reveal that in WTT PT cells most of the Ca^2+^ entry is through ROCE, whereas in KOT PT cells SOCE is the major conduit, particularly through IP_3_-induced Ca^2+^ release, as evidenced by greater 2-APB and Pyr6 inhibition on Ca^2+^ entry in KOT cells compared to WTT cells. Since SOCE can also be mediated by ER mediated STIM-ORAI pathway [24], however, store-depleted responses were not prominent in KOT PT cells. Acz is a known pH inducer, which raises the possibility that the Acz treated PT cells’ HCO_3_^−^ transporting activity would be higher. However, we found a compromised HCO_3_^−^ handling in Acz-treated PT cells and greater NBCe1 expression in the TRPC3 KO than in the WT, as well as greater activity. Perhaps the apical reclamation activity of HCO_3_^−^ was increased by another transporter [51].

Analyses of urine K^+^ levels following Acz treatment reveled that K^+^ levels were decreased while there is an increase in serum K^+^ (Figure S1). The observed differences in K^+^ clearance particularly for the treated groups provide explanation for the observed increase in urinary pH among the treated groups. Studies have shown that, Acz results in increased Na^+^ clearance which can be as a result of Acz’s inhibitory action on CA [45,52]. Similarly, Na^+^ excretion is directly related to decrease in K^+^ excretion which is associated with increasing serum K^+^ levels [53]. It is possible that elevated serum K^+^ levels are directly correlated to lower blood pH and higher urine pH due to the mechanism of intercalated cells [54], which we plan to consider in a future study. Higher urinary pH has been shown to be conducive for CaP crystal formation [55], which we found in both of our treated (KOT and WTT) groups. However, the elevated progression of CaP+CaOx (mixed) crystal formation in the KOT group showed the associated increase in urine CaP and the absence of TRPC3 provide evidence of the possible protective role of TRPC3 in calcium crystal formation possibly through its action in regulating the PT Ca^2+^ influx and preventing nephrocalcinosis. Besides PT, TRPC3 is also expressed in collecting duct [56], therefore renal Ca^2+^ clearance in PT-specific TRPC3 deleted mouse model may be required to sort out the contribution of PT in TRPC3-mediated urinary Ca^2+^ changes in response to Acz treatment.

Studies have shown the PT cells are much more prone to oxidative damage and thus the site for the pathogenesis of major kidney diseases [33]. Similarly, previous studies have shown that in the occurrence of PT damage and in kidney diseases, PT cells evoke inflammatory fibrosis [57]. Previous studies involving clinical cases of RTA have shown associations between RTA and inflammation with mild cases of fibrosis [58,59], all of which has been demonstrated in our Acz treated groups particularly among the KOT mice. Moreover, our findings of exacerbated fibrosis and inflammation in absence of TRPC3 can pave the way to link TRPC3 in renal calcification and inflammation/fibrosis. Chronic renal acidosis leads to increased oxidative activity particularly in the PT [60] which we have shown in our study that Acz not only increased the expression of ROS genes but upregulated the oxidative protective genes among the treated groups. Similarly, the increased expression levels of GPX3 and 6 in treated groups can be supported by the increases ROS levels and glutathione peroxidase activity on chronic renal failure and renal acidosis [60]. Moreover, elevated ROS production in PT can induce ER-stress leading to cellular damage and apoptosis [61]. Thus, the upregulation of M18S in the KOT particularly provides support for ER-stress induced apoptosis, and significantly, these results corroborate the significant role of TRPC3 in the PT and in the development of CKD. Furthermore, increased Ca^2+^ release for the treated groups may correlate with ER stress induction, which in turn increases ROS formation [62].

**Scheme 1:**
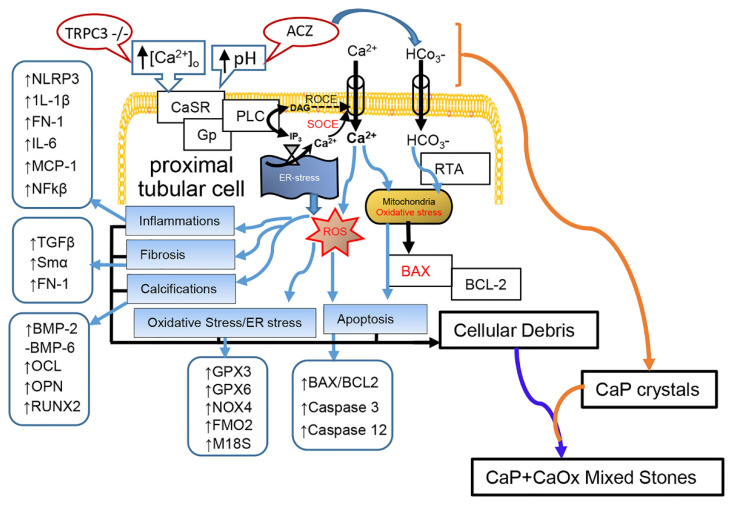
Proposed mechanism of TRPC3-/- mouse proximal tubular cell in alkaline conditions following acetazolamide treatment. Proximal tubular (PT) cell of moderately hypercalciuric (TRPC3-/-) mice after inducing alkalization of tubular pH with Acz treatment. Mode of Ca^2+^ entry in PT cells switches from receptor-operated Ca^2+^ entry (ROCE) to store-operated Ca^2+^ entry (SOCE) after being subjected to alkaline conditions. This also prompts bicarbonate current activation, facilitation of renal tubular acidosis, and favorable conditions for calcium phosphate (CaP) crystal formation in the tubular lumen. Such luminal alkalization activates detrimental pathways in PT cells, including: ROS generation, calcification, inflammation, and fibrosis. In turn, increased ROS formation leads to ER stress and apoptosis towards PT cell injury. Cellular debris from PT cells can bind to CaP crystals and increase the risk of CaP/CaOx renal stone formation. Calcification markers are Osteocalcin 2 (OCL2), Osteopontin 4 (OPN-4), and Runt-related transcription factor 2 (RUNX2), and bone morphogenic protein (BMP) 2 and 6. Inflammation markers are NFkβ, MCP1, NLRP3 and Interleukins (IL): IL-1β, IL-6; and fibrotic markers are SMa, TGFβ1, and FN-1. Acz: acetazolamide; RTA: renal tubular acidosis; CaSR: calcium sensing receptor; CaP: calcium phosphate; CaOx: calcium oxalate; Gp: G-protein; ROS: reactive oxygen species; ROCE: receptor-operated Ca^2+^ entry; SOCE: store-operated Ca^2+^ entry; ER: endoplasmic reticulum.

Here, we investigated the PT Ca^2+^ signaling mechanisms and the underlying pathophysiology of CaNL using mild hypercalciuric (TRPC3-/-) mice through alkalization of tubular pH using Acz to mimic the human-like mixed calcium stone pathophysiology (Scheme S1). We established that the mode of Ca^2+^ signaling signature in PT switched from ROCE to SOCE due to renal alkalization, which may be linked to significantly enhanced bicarbonate currents to induce CaP crystal formation. More importantly, the renal alkalization in TRPC3 KO mice intensified the pathophysiologic pathway activation of PT cell injury through ROS generation, calcification, inflammation, fibrosis, and apoptosis. Thus, higher luminal pH could be associated with intracellular acidosis and ER stress. Notably, PT cell injury in absence of TRPC3 can significantly predispose growth and aggregation of CaP crystals because damaged PT cellular debris, when binding to CaP crystals, can aggravate the process of stone formation (Scheme 1). Together, these results can help us to understand the link between alkalization-induced PT Ca^2+^ signaling and CaP crystal formation, which may have further stimulated CaP+CaOx mixed stones as found in clinical conditions of CaNL.

## 4. Materials and Methods

### 4.1. Animals 

TRPC3-/- and WT mice both were purchased from the Jackson Laboratory (Bar Harbor, ME, USA) and were maintained and crossed as described previously [18,63]. Importantly, use of mice in this study were approved through protocol designed in accordance with the Guiding Principles in the NIH Care and Use of Animals, approved by the Institutional Animal Care and Use Committee and the Research and Development Committee of DC Veterans Affair Medical Center. Both WT and TRPC3 KO mice were treated with oral Acz (0.08%) to induce alkalinurea and were designated as WTT and KOT respectively.

### 4.2. Materials, Antibodies and Chemicals 

Acz, L-Phe, Pyr6 and Pyr10 were purchased from Sigma-Aldrich (St. Louis, MO, USA) whereas Pyr3, 2-APB, NPS-2143, and SKF-96365 were purchased from Tocris Bioscience (Minneapolis, MN, USA). All cell culture media including Dulbecco’s modified Eagle’s medium (DMEM), fetal bovine serum (FBS), antibiotics (penicillin and streptomycin), glutamine, and Fura-2/acetoxymethyl ester (AM) were purchased from Invitrogen (Carlsbad, CA, USA). All other chemicals used in this study were analytical grade. 

### 4.3. Mice Urine and Serum Electrolyte and pH Measurements

Ad libitum fed mice were housed in their respective metabolic cages (Nalgene, Rochester, NY, USA) for 24 h urine collection. WT, WTT, KO, and KOT mice treatment groups cages were designated for treatment with drinking water containing 2% sucrose solution alone (WT, KO) or with 0.08% Acz (WTT, KOT). pH was measured immediately after collection using Orion Star A121 portable pH meter (Thermo Fisher Scientific, Waltham, MA, USA) [64]. Urine and serum electrolytes (Na^+^, K^+^, and Cl^−^) were measured using a Medica EasyLyte Analyzer (Bedford, MA, USA). Urine and serum Ca^2+^ measurements were confirmed with the Randox Calcium Assay Kit (Randox Laboratories, Kearneysville, WV, USA). Urine and serum PO_4_^3−^ were measured using a QuantiChrom™ Phosphate Assay Kit (BioAssay Systems, Hayward, CA, USA). Food and water intake were recorded throughout the period.

### 4.4. Alizarin Red Staining of Urine Crystals

AR staining was performed to detect the presence of CaP and/or CaOx crystals in mice urine as described previously [15,65,66,67]. Urine was briefly centrifuged, then crystals and cells were subsequently collected. Equal volumes of AR pH 4.3 (stains CaP crystals) or pH 6.8 (stains both CaP and CaOx crystals) were added to the crystals, then incubated at 37 °C for 30 min. Afterwards, supernatants were removed and those crystal pellets were mounted in slides for capturing images for stained crystals using light microscopy with Zeiss Axio Observer.Z1 Microscope. Stained images were quantified using ImageJ as previously described [68].

### 4.5. Isolation and Primary Culture of PT Cells

PT cells from WT, WTT, KO, or KOT mice were isolated and cultured as previously described [18]. Briefly, kidneys were decapsulated immediately after extraction and washed with cold external solution containing 0.02% soybean trypsin inhibitor and 0.1% bovine serum albumin. The cortical tissues were used to obtain PT cells through an enzymatic digestion buffer containing 1% Worthington collagenase Type IV and 0.25% soybean trypsin inhibitor. Cells were passed through nylon mesh (pore size 70µm) filter and then resuspended to centrifugation at ~27,000× *g* for 10 min at 4 °C. The cells were washed with media (DMEM containing 10% FBS, supplemented with 2 mmol/L glutamine, 100 IU/mL penicillin, 100 µg/mL streptomycin, 5 µg/mL insulin, 5 × 108 mol/L hydrocortisone, 5 µg/mL transferring, 2 mmol/L butyrate, 2 mmol/L alanine, and 2 mmol/L lactate supplementation), and were immediately used for electrophysiology experiments as described previously [18] or cultured as described [69] for Ca^2+^ imaging experiments.

### 4.6. Time-Lapse [Ca^2+^]_i_ Fluorescence Measurements

As previously described, time-dependent ratiometric (340/380) [Ca^2+^]_i_ measurements of PT cells from WT, WTT, KO, or KOT mice were performed [62,70]. Cells loaded with Fura-2 were placed in a microincubator set to 37 °C along with a gaseous mixture of 95% air and 5% CO_2_ for the duration of the experiment. Cell images were taken using an IX81 motorized inverted microscope equipped with an IX2-UCB control box (Olympus USA, Center Valley, PA, USA). These images were fed into a C9100-02 electron multiplier CCD camera with an AC adaptor A3472-07 (Hamamatsu, Bridgewater, NJ, USA). A Lambda-LS xenon arc lamp and a 10-2 optical filter changer (Sutter Inst. Novato, CA, USA) was added as an illuminator which can output 340 and 380 nm to a 700 nm cutoff. Fluorescence emitted by Fura-2 is excited at a wavelength of 500 nm with peak absorbance continuously shifting at wavelengths of 340 nm and 380 nm. PT cells were brought into focus using a differential interference contrast channel. These measurements were digitally processed using the 3i SlideBook version 5.0 microscopy software (Intelligent Imaging Innovations, Denver, CO, USA). Time-lapse was configured to 200–500 time points at 1 sec intervals, and 50–150 cells were selected as regions of interest (background fluorescence automatically subtracted prior to 340/380 ratio calculation and graphing). Analysis was performed offline using Slidebook^TM^ software.

### 4.7. Whole-Cell Patch Clamp Experiments

Electrophysiology were performed on each single cell and the ion currents were measured using whole-cell patch clamp technique as previously described [62,71]. Typically, all extracellular solution surrounding a cell were comprised of (in mM): 140 NaCl, 4 KCl, 1 MgCl_2_, 2 CaCl_2_, 5 D-glucose and 10 HEPES (NaOH, pH 7.4). Intracellular solution comprised of (in mM): 50 CsCl, 10 NaCl, 60 CsF, 20 EGTA, and 10 HEPES (CsOH, pH 7.2). Whole-cell voltage ramp recordings were performed using EPC-10 digitally controlled amplifier and Patchmaster software (HEKA, Lambrecht, Germany). Data was obtained at 5.00 kHz and filtered with 2.873 kHz. Current-voltage (I-V) curves were outputted every 3 s after application of voltage ramps (300 ms) from −100 mV to +100 mV, with a holding potential of −80 mV. Once the whole-cell configuration achieved the cut off resistance was established as >500 MΩ. All experiments were performed at 25 °C.

### 4.8. RNA Extraction, cDNA Synthesis and Polymerase Chain Reaction (PCR)

Total RNAs were isolated from mouse PT cells using TRIzol reagent as previously described [62]. DNase and cDNA synthesis were performed using GoScript™ Reverse Transcription System (Promega, Madison, WI, USA) as per the manufacturer’s instructions. Gene amplification was performed using gene-specific primers (Table 1) and PCR Master Mix prepared using GoTaq^®^ Green Master Mix (Promega, Madison, WI, USA). Primers were purchased from Invitrogen and Integrated DNA Technologies (Coralville, IA, USA). Thermocycling (Bio-Rad, Hercules, CA, USA) parameters were set to PCR conditions as previously described [62].

### 4.9. Protein Analysis and Western Blotting

Protein preparation, SDS-PAGE (using 4–12% gels; Invitrogen), transferred from the gel onto an Immobilon-PSQ transfer membrane (Millipore Corp, Bedford, MA) were performed at room temperature as previously described [18,70]. Antibody labeling was detected by enhanced chemiluminescence (ECL) using primary antibodies: mouse monoclonal antibodies (Table 2). Anti-mouse secondary antibodies from Sigma-Aldrich and ECL kit from Pierce (Invitrogen) were used.

### 4.10. Histochemistry of Kidney Sections

Kidneys collected from the mice after euthanasia were immediately fixed in 10% formalin solution for 24 h and then dehydrated in graded concentrations of ethanol and then embedded in paraffin. Mice tissue sections (∼5–7 µm) were prepared from whole kidney tissue paraffin blocks. Histochemistries were performed on paraffin sections of treated mice kidneys, as described previously [72]. Sections were stained with H&E, Masson’s, Von Kossa or AR pH 4.3 or pH 6.8 as described previously [73].

### 4.11. Statistical Analysis 

Origin 6.1 was used as a graphical software for analyses of experimental data such as plotting, and curve-fitting, etc. The data were expressed as means ± SEM. To obtain statistically valid data comparisons were made using Student’s unpaired *t*-test (two-tailed), or ANOVA, as appropriate, in Origin 6.1. Statistically significant comparisons were accepted at *p* < 0.05.

## Figures and Tables

**Table 1:** PCR Primer information.

Primer	Sequence (Sense, Antisense)	Product Size (bp)
mGAPDH	5′-ACTCCACTCACGGCAAATTC-3′	171
	5′-TCTCCATGGTGGTGAAGACA-3′	
mNCX1	5′-CCTTGTGCATCTTAGCAATG-3′	309
	5′-TCTCACTCATCTCCAGA-3′	
mPMCA1	5′TGGCAAACAACTCAGTTGCATATAGTGG3′	562
	5′TCCTGTTCAATTCGACTCTGCAAGCCTCG3′	
mSMa	5′-AGATTGTCCGTGACATCAAGG-3′	538
	5′-TTGTGTGCTAGAGGCAGAGC-3	
mNBCe1	5′-CACTGAAAATGTGGAAGGGAA-3′	544
	5′-TTATCACCCTTGTGCTTTGC-3′	
mNaPiIIa	5′-AGACACAACAGAGGCTTC-3′	181
	5′-CACAAGGAGGATAAGACAAG-3′	
mBMP2	5′-TGGAAGTGGCCCATTTAGAG-3′	166
	5′-TGACGCTTTTCTCGTTTGTG-3′	
mBMP6	5′-CCCGCCCGGAGTAGTTTTAGC-3′	168
	5′-AGTGCCCTTCTCCCCTCCATT-3′	
mOCL	5′-CTGACAAAGCCTTCATGTCCAA-3′	59
	5′-GCGCCGGAGTCTGTTCACTA-3′	
mOPN	5′-GATGATGATGACGATGGAGACC-3′	148
	5′-CGACTGTAGGGACGATTGGAG-3′	
mRUNX2	5′-CGGCCCTCCCTGAACTCT-3′	75
	5′-TGCCTGCCTGGGATCTGTA-3′	
mTGFb	5′-CTGAGTGGCTGTCTTTTG-3′	436
	5′-TTGCTGTACTGTGTGTCC-3′	
mFN1	5′-TGCACGATGATATGGAGAGC-3′	93
	5′-TGGGTGTCACCTGACTGA-3′	
mNLRP3	5′-AGAGCCTACAGTTGGGTGAAATG-3′	116
	5′-CCACGCCTACCAGGAAATCTC-3′	
mIL-1b	5′-TCCATGAGCTTTGTACAAGGA-3′	343
	5′-AGCCCATACTTTAGGAAGACA-3′	
mIL-6	5′-TGGAGTCACAGAAGGAGTGGCTAA-3′	155
	5′-TCTGACCACAGTGAGGAATGTCCA-3′	
mMCP1	5′-AGAGAGCCAGACGGGAGGAA-3′	520
	5′-GTCACACTGGTCACTCCTAC-3′	
mNFkb	5′-GTGGAGGCATGTTCGGTAGT-3′	367
	5′-AGCTGCAGAGCCTTCTCAAG-3′	
mGPX3	5′-TGGCTTGGTCATTCTGGGC-3′	103
	5′-CCCACCTGGTCGAACATACTT-3′	
mM18S	5′-ACGGAAGGGCACCACCAGGA-3′	127
	5′-CACCACCACCCACGGAATCG-3′	
mBAX	5′-GAGACACCTGAGCTGACCTT-3′	244
	5′-GCACCAGTTTGCTAGCAAAG-3′	
mBCL2	5′-CTCGTCGCTACCGTCGTGACTTCG-3′	242
	5′-CAGAGTCCGGTTCAGGTACTCAGTC-3′	
mCaspase3	5′-AGAGAGCCAGACGGGAGGAA-3′	519
	5′-GTCACACTGGTCACTCCTAC-3′	
mCaspase12	5′-GCTGGCCACATTGCCAATTCCC-3′	314
	5′-GCCAGACGTGTTCGTCCCTCC-5′	
mGPX6	5′-GCCCAGAAGTTGTGGGGTTC-3′	129
	5′-TCCATACTCATAGACGGTGCC-3′	
mSLC26a	5′-AGATCTTCCTTGCGTCTGC-3′	149
	5′-GCCTTTCCACATGGTAGTCTC-3′	

**Table 2:** Antibody information.

Antibody	Company Purchased/Ref. Number	Dilution Used
BAX1	Santa Cruz Biotechnology; sc-20067	1:1000
BCL2	Santa Cruz Biotechnology; sc-23960	1:1000
Caspase-3	Santa Cruz Biotechnology; sc-7272	1:1000
OPN	Santa Cruz Biotechnology; sc-10591	1:1000
RUNX2	Santa Cruz Biotechnology; sc-101145	1:1000
BMP2	Santa Cruz Biotechnology; sc-6895	1:1000
BMP6	Santa Cruz Biotechnology; sc-7406	1:1000
OCL	Santa Cruz Biotechnology; sc-376726	1:1000
Beta Actin	Santa Cruz Biotechnology; sc-47778	1:1000
SLC4A4 (NBCe1)	Proteintech; 11885-1-AP	1:1000

## Data Availability

The data that support the findings of this study are available from the corresponding author upon reasonable request.

## Supplementary Materials

The following are available online at https://www.mdpi.com/1422-0067/22/6/3050/s1.
