# Supplementary material for: Modulation of Tubular pH by Acetazolamide in a Ca2+ Transport Deficient Mice Facilitates Calcium Nephrolithiasis
Source: Int J Mol Sci. 2021 Mar 17;22(6):3050. doi: 10.3390/ijms22063050 (PMC8002449; doi:10.3390/ijms22063050)
Supplement: Supplementary file 1 [file ijms-22-03050-s001.pdf]

SUPPLEMENTAL MATERIALS

Modulation of tubular pH by acetazolamide in a Ca^{2+} transport deficient mice facilitates calcium nephrolithiasis

Eugenia Awuah Boadi[1], Samuel Shin[1], Samuel Yeroushalmi[1,2], Bok-Eum Choi[1], Peijun Li[1] and Bidhan C. Bandyopadhyay[1,2,3*]

[1] Calcium Signaling Laboratory, Research Service, Veterans Affairs Medical Center, 50 Irving Street, NW, Washington, DC 20422, USA
[2] Division of Renal Diseases & Hypertension, Department of Medicine, The George Washington University, Washington DC, 20037, USA
[3] Department of Biomedical Engineering, The Catholic University of America, 620 Michigan Avenue NE, Washington DC, 20064, USA.

Running title: Tubular alkalization induces kidney stones

Keywords: Acetazolamide; renal tubular pH; proximal tubule; Ca^{2+} signaling; oxidative stress; inflammation; fibrosis; apoptosis; calcium nephrolithiasis; and urinary stones.

[*]**Corresponding author**: Bidhan C. Bandyopadhyay, PhD. Chief, Calcium Signaling Laboratory, 151 Research Service, Veterans Affairs Medical Center, 50 Irving Street, NW, Washington, DC 20422, Phone: (202) 745-8622, Fax: (202) 462-2006; E-mail: bidhan.bandyopadhyay@va.gov

ORCID IDs:

Eugenia Awuah Boadi: https://orcid.org/0000-0002-2858-4875

Samuel Shin: https://orcid.org/0000-0002-3461-2844

Samuel Yeroushalmi: https://orcid.org/0000-0002-8327-3279

Bok-Eum Choi: https://orcid.org/0000-0002-8154-1292

Peijun Li: https://orcid.org/0000-0003-0701-3122

Bidhan C. Bandyopadhyay: https://orcid.org/0000-0003-2364-8945

Supplemental Figures

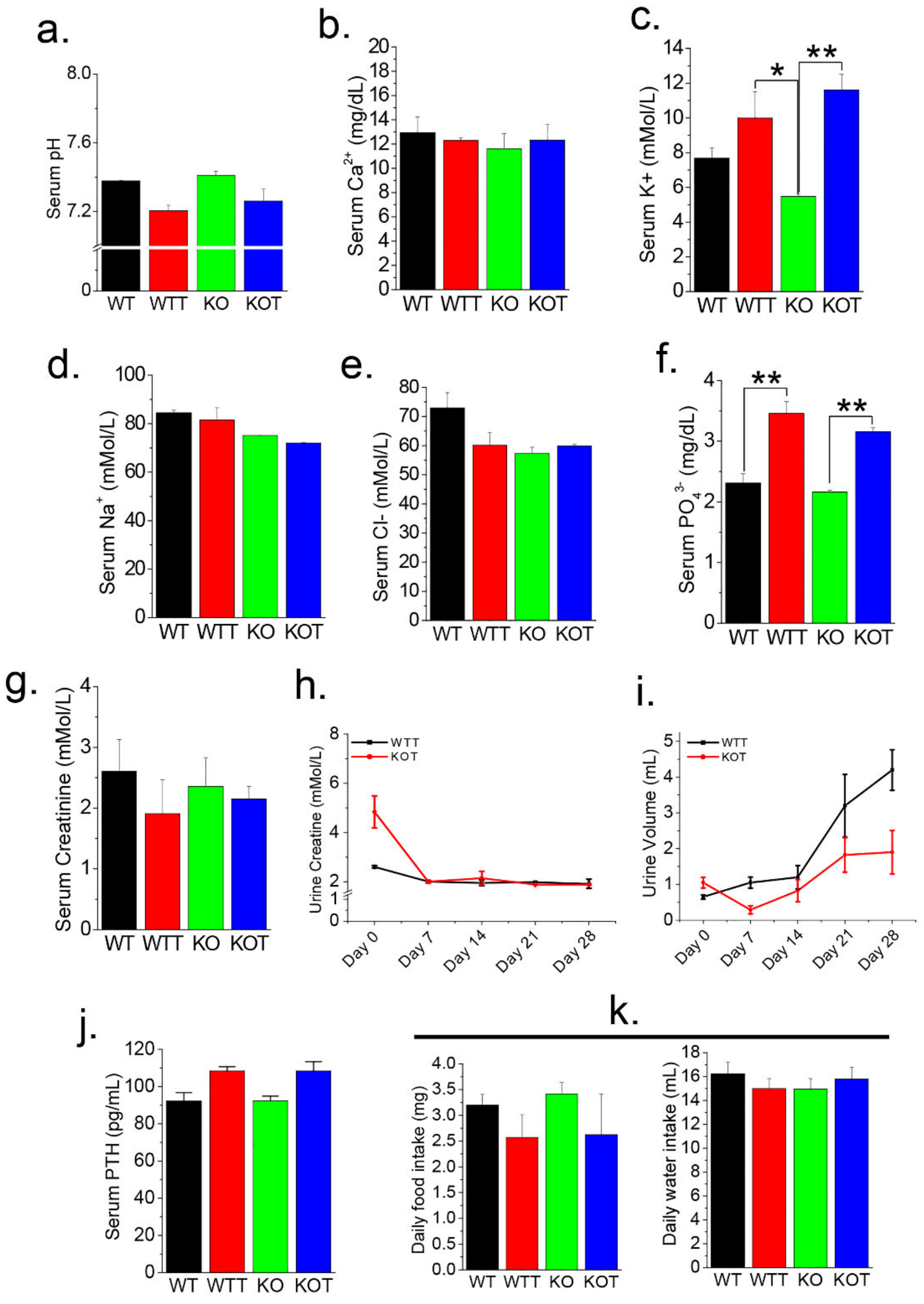

Figure S1. Serum pH, Electrolytes and PTH; Urine Creatine and volume; and daily Ca^{2+}, food and water intake measurements and gene expression of mice PT transepithelial markers following Acz Treatment. **(a)** Serum pH, average serum electrolytes levels of **(b)** Ca^{2+}, **(c)** K^{+}, **(d)** Na^{+}, **(e)** Cl^{-}, **(f)** PO_4^{3-} and **(g)** Creatine. Except for serum K^{+} **(c)**, all serum electrolytes did not show any significant changes between the untreated group (WT and KO) and the treated group (WTT and KOT). **(h)** Urine creatinine and **(i)** Urine volume. Urine creatine levels remained constant throughout the treatment period while urine volume showed a progressive increase with treatment period, showing the diuretic effect of Acz. **(j)** Serum PTH measurements show no significant changes among the groups. Average daily intake of **(k)** food and water. Representative bar graphs are plotted from means ±SEM of 4 separate experiments. *, $p < 0.05$; **, $p < 0.01$.

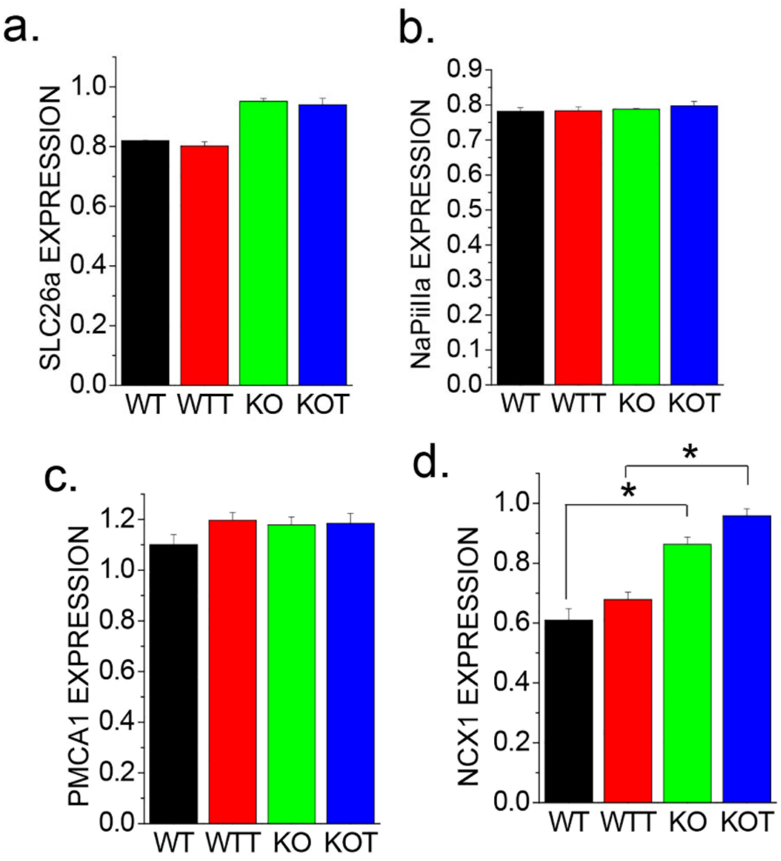

Figure S2. Gene expression analysis of transcellular markers were performed for **(a)** SLC26a, **(b)** NaPiIIa, **(c)** PMCA1 and **(d)** NCX1. All transcellular machinery makers were normalized to GAPDH. Assessment of the transcellular markers showed no significant difference between the mice types (WT, WTT and KO, KOT) except for NCX1 which showed increased expression in the KO/KOT groups. Representative bar graphs are plotted from means ±SEM of 4 separate experiments. *, $p < 0.05$; **, $p < 0.01$.

Supplemental Diagram

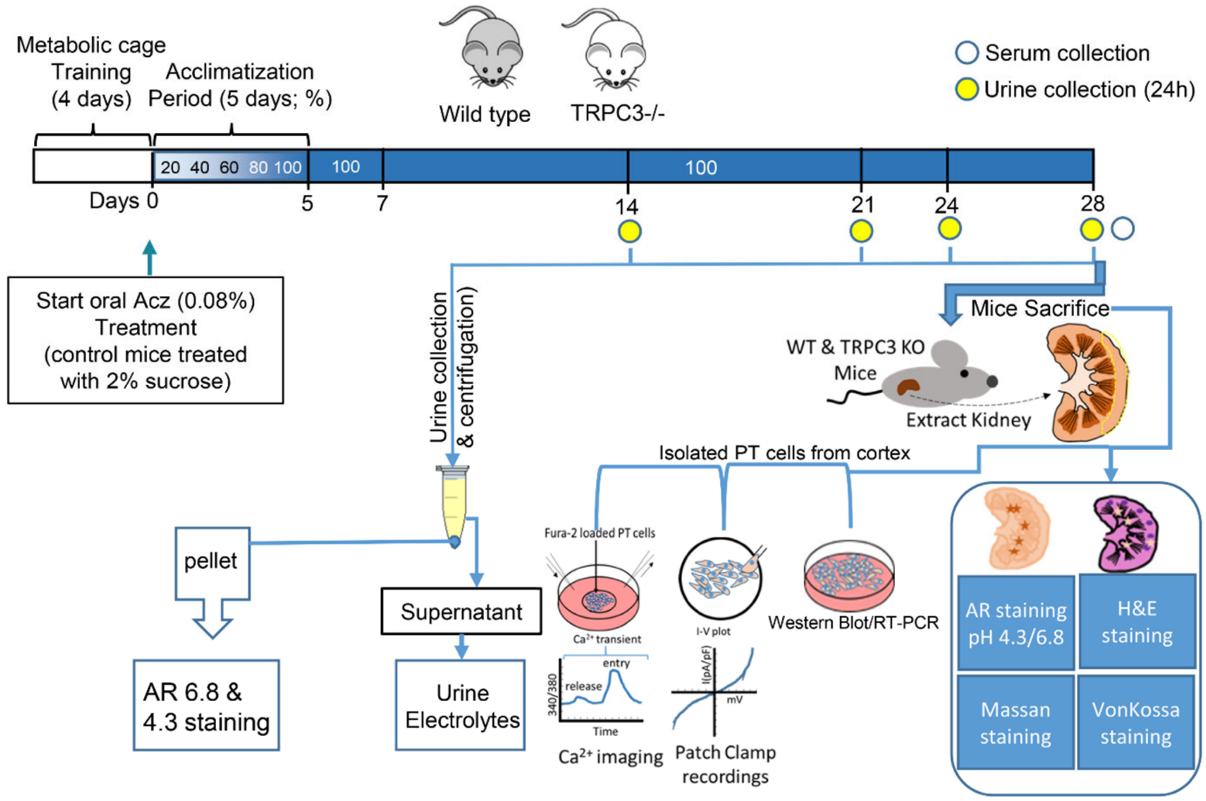

Supplemental Diagram 1. Treatment protocol and experimental methodology. WT and TRPC3-/- mice were acclimatized with 0.08% acetazolamide (Acz) followed periodic 24h urine collection and subsequent centrifugation to separate pellet (for alizarin red pH 4.3/6.8 staining) and supernatant (for urinalysis). At the end of treatment, all mice were sacrificed, and serum was collected (for PTH and electrolyte analysis). Following sacrifice, kidneys were extracted (for fixing, embedding, sectioning and alizarin red (pH4.3/pH6.8, H&E, Masson's, or Von Kossa staining) and proximal tubular cells were isolated from the cortex (for Ca^{2+} imaging, whole-cell patch clamp recordings, western blot, or RT-PCR).
